# Influence of Substrate Manufacturing Route on HiPIMS TiAlSiN-Coated AISI 316L Stainless Steel Produced by Laser Powder Bed Fusion

**DOI:** 10.3390/ma19061184

**Published:** 2026-03-18

**Authors:** Marek Kočiško, Patrik Petroušek, Róbert Kočiško, Lukáš Štafura, Dávid Medveď, Róbert Džunda

**Affiliations:** 1 Faculty of Manufacturing Technologies, Technical University of Kosice, Bayerova 1, 080 01 Presov, Slovakia; lukas.stafura@student.tuke.sk; 2 Institute of Materials, Faculty of Materials, Metallurgy and Recycling, Technical University of Kosice, Letná 1/9, 042 00 Košice, Slovakia; robert.kocisko@tuke.sk; 3 Institute of Materials Research, Slovak Academy of Sciences, Watsonova 47, 040 01 Košice, Slovakia

**Keywords:** laser powder bed fusion, AISI 316L stainless steel, HiPIMS, TiAlSiN coating, mechanical properties, microstructure

## Abstract

Laser powder bed fusion has attracted increasing attention for the production of metallic substrates intended for surface functionalization by advanced physical vapor deposition coatings. This study investigates the influence of the substrate manufacturing route on the performance of titanium–aluminum–silicon nitride-coated AISI 316L stainless steel, with particular emphasis on substrates produced by laser powder bed fusion. Conventionally manufactured and additively manufactured AISI 316L substrates were coated with a titanium–aluminum–silicon nitride layer using high-power impulse magnetron sputtering. The substrates were characterized by tensile testing and microhardness measurements, while coating thickness and uniformity were evaluated using the crater ball method. The mechanical integrity of the coating–substrate system was assessed by progressive load scratch testing. The additively manufactured substrate exhibited a significantly higher yield strength (411 MPa) compared to the conventionally manufactured material (257 MPa), together with increased microhardness. The titanium–aluminum–silicon nitride coating showed a uniform thickness of 4.47 µm and a well-defined coating–substrate interface. Scratch tests revealed a delayed onset of coating damage on additively manufactured substrates, with the transition to severe adhesive failure occurring at higher normal loads compared to the conventionally manufactured substrate. These results demonstrate that AISI 316L stainless steel produced by laser powder bed fusion provides a mechanically robust substrate for titanium–aluminum–silicon nitride coatings deposited by high-power impulse magnetron sputtering, with favorable coating response under progressive loading conditions.

## 1. Introduction

Surface engineering represents one of the key areas of modern mechanical engineering, as it enables targeted enhancement of functional properties of components without modifying their bulk microstructure. The application of surface coatings is a well-established approach for improving wear resistance, reducing friction, and extending the service life of components exposed to mechanical and tribological loading [1,2,3,4]. In tooling and general functional applications, research therefore focuses primarily on hard PVD coatings and low-friction DLC systems, which allow stable operational performance even under demanding contact conditions [5].

Among advanced hard coatings, nanocomposite nitride systems such as (Al,Ti,Si)N are considered highly promising due to their high hardness and thermal stability, which arise from their characteristic nanocrystalline–amorphous microstructure [6,7]. However, the resulting properties of these coatings are not determined solely by their chemical composition but are strongly influenced by the deposition technology employed. In recent years, increasing attention has therefore been paid to comparisons between conventional cathodic arc PVD (Arc PVD) and advanced high-power impulse magnetron sputtering (HiPIMS) technologies, which provide a higher degree of ionization of the depositing species and consequently lead to different coating growth mechanisms [8,9,10]. HiPIMS-deposited TiAlSiN coatings typically exhibit a nanocomposite structure consisting of TiAlN nanocrystalline grains embedded in an amorphous SiNx matrix. This architecture leads to high hardness (typically ~30–35 GPa) and enhanced thermal stability due to the inhibition of grain growth by the amorphous SiNx phase [11,12,13].

Saciotto et al. [14] demonstrated that AlTiN coatings deposited by HiPIMS exhibit a denser microstructure, higher hardness, and improved adhesive behavior compared to coatings produced by Arc PVD. These differences were attributed to the higher energy of impinging ions and more effective filling of microdefects during coating growth. Similar conclusions were reported by work [9], who analyzed the fundamental physical principles of HiPIMS deposition and highlighted its potential for producing coatings with high layer integrity and favorable mechanical properties. However, most of these studies primarily focus on the coating system and deposition process itself, while the influence of the substrate manufacturing route on coating performance remains largely a secondary consideration [15,16].

In addition to hard nitride coatings, extensive research has been devoted to diamond-like carbon (DLC) coatings, which are characterized by a low coefficient of friction and favorable tribological behavior. The fundamental mechanisms governing DLC coatings, as well as the influence of hydrogen content and metallic dopants, were comprehensively described by works [17,18]. For engineering applications, modified DLC systems such as WC-C:H coatings with appropriately designed multilayer architectures and chromium-based interlayers are frequently employed to improve adhesion to steel substrates. Authors Bewilogua and Hofmann [19] emphasized that coating architecture and the quality of the coating–substrate interface are decisive factors governing the mechanical stability of DLC coatings. Podgornik et al. [20] further reported that, in W-DLC systems, the condition of the substrate and its surface integrity have a pronounced effect on damage mechanisms and the overall tribological response of the coating.

Alongside the rapid development of coating technologies, additive manufacturing of metallic components—particularly Direct Metal Laser Sintering (DMLS/LPBF)—has gained significant attention in recent years. The materials produced by additive manufacturing exhibit specific microstructural characteristics that differ markedly from those of conventionally manufactured materials. In the case of austenitic stainless steel AISI 316L, these characteristics include a fine cellular microstructure, anisotropy of mechanical properties, residual stresses, and the presence of porosity. These features directly affect the so-called surface integrity of the material, which plays a crucial role in coating deposition and performance [21,22,23,24,25].

Several studies have already addressed the coating of additively manufactured 316L, highlighting the importance of surface finishing and its influence on coating adhesion and failure mechanisms [26]. Nevertheless, Bonnici et al. [27] concluded in their review that the literature dealing with PVD and DLC coatings applied directly to additively manufactured 316L remains limited and fragmented. The authors further emphasized the lack of systematic studies directly comparing conventionally manufactured and additively manufactured 316L substrates under identical deposition and testing conditions. From the perspective of adhesion assessment, scratch testing is widely recognized as one of the fundamental and standardized experimental methods for evaluating coating performance [28].

From a practical application standpoint, the functional performance of coated components is governed not only by the coating composition itself but also by the coating–substrate interaction, which may be significantly influenced by the manufacturing route of the base material [29].

Based on the reviewed literature, it can be stated that comprehensive and directly comparable experimental studies evaluating coatings deposited on conventionally manufactured and additively manufactured AISI 316L under identical conditions remain limited. In particular, systematic investigations focusing on the influence of the substrate manufacturing route on the performance of a single coating system are scarce. Consequently, the extent to which differences in substrate microstructure and surface integrity affect coating adhesion, hardness, damage mechanisms, and tribological performance has not yet been fully clarified. The objective of this study is therefore to experimentally evaluate the properties of a nanocomposite AlTiSiN coating deposited on conventionally manufactured and additively manufactured (polished) AISI 316L substrates. The coating is prepared using HiPIMS. The evaluation focuses on coating microstructure, mechanical properties, adhesive behavior, and damage mechanisms identified through scratch testing and related analytical techniques, with the aim of assessing the influence of the substrate manufacturing route on the mechanical integrity and damage response of the coating–substrate assembly.

## 2. Materials and Methods

### 2.1. Base Material and Sample Manufacturing

The investigated material was austenitic stainless steel AISI 316L (EN 10088-1 [30]), selected due to its widespread use in engineering applications and its suitability for both conventional and additive manufacturing routes. Two types of substrates were examined in this study: conventionally manufactured 316L steel and additively manufactured 316L steel produced by laser powder bed fusion (LPBF).

The additively manufactured samples were fabricated by LPBF (also referred to as Selective Laser Melting, SLM) using a DMG MORI LASERTEC 30 SLM (DMG MORI Additive GmbH, Bielefeld, Germany) system equipped with a single fiber laser with a maximum power of 600 W. The manufacturing process was carried out in an inert argon atmosphere, with oxygen removed from the build chamber prior to the start of melting to minimize oxidation during processing. The build direction was oriented perpendicular to the base plate, and a layer thickness of 0.05 mm was applied. The tensile specimens were manufactured with the loading direction parallel to the build direction. The ambient temperature during fabrication was approximately 25 °C.

The LPBF process parameters used for the fabrication of AISI 316L samples are summarized in Table 1. The selected parameters correspond to typical optimized processing conditions for LPBF-produced 316L stainless steel and result in dense parts with mechanical properties characteristic of additively manufactured AISI 316L. The corresponding volumetric energy density was approximately 56 J·mm^−3^.

The applied LPBF process complied with the requirements of ISO/ASTM 52900 and ISO/ASTM 52910 standards [31,32].

As feedstock material, gas-atomized austenitic stainless steel powder AISI 316L supplied by Thyssenkrupp Materials was used. The chemical composition of the powder corresponded to EN 10088-3 [33] (X2CrNiMo17-12-2), with a typical composition of 16.5–18.5 wt.% Cr, 10–13 wt.% Ni, 2.0–2.5 wt.% Mo, Mn ≤ 2 wt.%, Si ≤ 1 wt.%, and C ≤ 0.03 wt.%. The powder exhibited predominantly spherical particle morphology and a particle size distribution suitable for LPBF processing, with an average particle size (D50) in the range of 30–35 μm, ensuring good flowability and stable powder spreading during fabrication.

The LPBF-fabricated samples were investigated in the as-built condition, without any post-process heat treatment. The only thermal exposure of the additively manufactured substrates occurred during subsequent coating deposition, where the substrate temperature did not exceed 510 °C. This approach allowed the intrinsic microstructural characteristics of the as-built LPBF material to be preserved while enabling coating deposition.

The conventionally manufactured AISI 316L steel used for comparison was supplied in a wrought condition after recrystallization and homogenization annealing, resulting in a typical fully austenitic microstructure with annealing twins. This material served as a reference representing the traditional production route.

Both conventionally manufactured and additively manufactured samples were subjected to identical surface preparation prior to subsequent characterization and coating deposition to minimize the influence of surface roughness and isolate the effect of the substrate manufacturing route on the investigated properties.

### 2.2. Surface Preparation and Coating Deposition

Prior to coating deposition, all samples manufactured from conventionally produced and additively manufactured AISI 316L stainless steel were prepared to ensure a comparable surface condition of the substrates. The sample surfaces were finished to a polished state using standard metallographic procedures in order to minimize the influence of surface irregularities originating from the manufacturing process. After polishing, the surface roughness of the polished substrates was measured using a contact profilometer Surftest SJ-301 (Mitutoyo Corp., Kawasaki, Japan) according to EN ISO 4287:1997 [34] (stylus radius 2 μm, cutoff length 0.8 mm). Twelve measurements were performed across the polished surfaces. The average roughness values after polishing were Ra = 0.553 ± 0.025 μm and Rz = 4.02 ± 0.32 μm, indicating a comparable final surface state prior to coating deposition.

Immediately before coating deposition, plasma surface pretreatment was carried out inside the coating chamber in an inert argon atmosphere. This pretreatment was applied to remove residual oxide layers and adsorbed contaminants from the substrate surface and to increase the surface energy, thereby promoting coating nucleation and adhesion. Such plasma activation represents a standard step in PVD and HiPIMS coating processes.

The coating was deposited using High Power Impulse Magnetron Sputtering (HiPIMS) on an industrial coating system. A nanocomposite TiAlSiN coating with a thickness of approximately 4.5 μm was deposited at 500 ± 10 °C, with a total thermal exposure of approximately 4.5 h, including substrate heating, Ar plasma pretreatment, and coating deposition. Both conventionally manufactured and additively manufactured substrates were coated simultaneously in a single deposition batch under identical processing conditions.

### 2.3. Microstructural Characterization, Porosity Evaluation, Fractography and DSC Technique

Microstructural characterization of conventionally manufactured and additively manufactured AISI 316L samples was performed by optical microscopy (OM) on polished and etched metallographic cross-sections. Observations were carried out using a Zeiss Axiovert A1 optical microscope (Carl Zeiss Microscopy GmbH, Jena, Germany) to identify the principal microstructural features associated with the different manufacturing routes.

Porosity evaluation was conducted on optical micrographs obtained by OM using digital image analysis. Image analysis was performed using the open-source software ImageJ (Version 1.54g, National Institutes of Health, Bethesda, MD, USA). The area fraction of pores was quantified to determine the total porosity of the material, enabling comparison of pore content between conventionally manufactured and additively manufactured samples. Porosity was evaluated on representative OM cross-sections prepared in the XY, Z, and 45° directions. For each orientation, 10 micrographs were analysed using ImageJ software. The pore area fraction was determined from individually evaluated OM images based on manual identification of pore regions. A more detailed description of the porosity determination procedure is provided in our previous study [21].

Fractographic analysis of selected fracture surfaces after mechanical testing was performed using scanning electron microscopy (SEM). The observations were carried out on a JEOL JSM-7000F scanning electron microscope (JEOL Ltd., Tokyo, Japan) to examine the fracture surface morphology and failure characteristics.

The thermal behavior of the investigated material was studied by simultaneous thermal analysis (STA) employing the differential scanning calorimetry (DSC) technique. Measurements were performed using an STA 449 F1 Jupiter system (NETZSCH, Waldkraiburg, Germany) with a constant heating rate of 30 K·min^−1^ from room temperature up to 700 °C. The analysis was carried out on as-built LPBF and LPBF-coated specimens with a mass of approximately 35 mg, which were placed in corundum crucibles and heated under a high-purity nitrogen atmosphere.

### 2.4. Mechanical Testing and Microhardness

Static tensile tests were performed on conventionally manufactured and additively manufactured AISI 316L samples at ambient temperature. The tests were carried out in accordance with EN ISO 6892-1 [35], Method B, using a TINIUS OLSEN H300KU universal testing machine (Tinius Olsen, Horsham, PA, USA). For each investigated condition, three tensile specimens were tested. From the recorded stress–strain curves, the yield strength (YS), ultimate tensile strength (UTS), total elongation (TE), and reduction in area (RA) were evaluated.

Microhardness measurements were conducted on polished sample surfaces using the Vickers method with a Struers Duramin-5 hardness tester (Struers A/S, Ballerup, Denmark) in accordance with ISO 6507-1 [36]. The measurements were performed at a load of HV0.5. For each sample, the reported microhardness values represent the average of multiple indentations distributed across the surface. The spacing between individual indentations exceeded 3.5 times the diagonal length of the indentation, thereby avoiding interference of the plastically deformed zones.

### 2.5. Tribological and Wear Testing

Scratch tests were performed to evaluate the adhesion and damage behavior of the HiPIMS-deposited coating using a CETR UMT tribometer (Bruker Corporation, Billerica, MA, USA). A Rockwell-type diamond indenter with a spherical tip was used as the counter body. The tests were conducted under progressive normal loading, with the normal force linearly increased from a low initial load to a maximum load of 180 N over a scratch length of approximately 6 mm. The normal load increased linearly over a duration of approximately 60 s, corresponding to a loading rate of about 3 N·s^−1^. The scratch speed was set to 0.1 mm·s^−1^. During the tests, the normal and tangential forces were continuously measured as a function of scratch length, and the coefficient of friction was obtained from the F_t_/F_z_ ratio. The critical loads corresponding to the onset of coating damage and coating failure were determined from changes in the frictional response and correlated with post-test OM observations of the scratch track. Three independent scratch tracks were performed for each condition, and the reported critical loads represent mean values ± standard deviation. The damage mechanisms were further analyzed by OM to distinguish between cohesive cracking and adhesive failure of the coating. Scratch tests were performed on polished surfaces corresponding to the build plane.

The coating thickness was determined using the crater ball (Calotest) method in accordance with EN 1071-6 [37]. The test was performed using an Olympus MX51 (Olympus, Tokyo, Japan) optical microscope equipped for calotte evaluation. A steel ball with a diameter of 20 mm was used as the counter body, while an abrasive suspension with a particle size of 0.5–1.0 µm (Struers, Ballerup, Denmark) was applied during the test. The ball rotation speed was set to 900 min^−1^, and the grinding time was 300 s. After testing, the resulting wear calotte was examined optically, and the inner and outer diameters of the calotte were measured. The coating thickness was calculated from the calotte geometry based on the measured diameters, assuming a spherical wear profile.

## 3. Results

### 3.1. Microhardness

**Figure 1:**
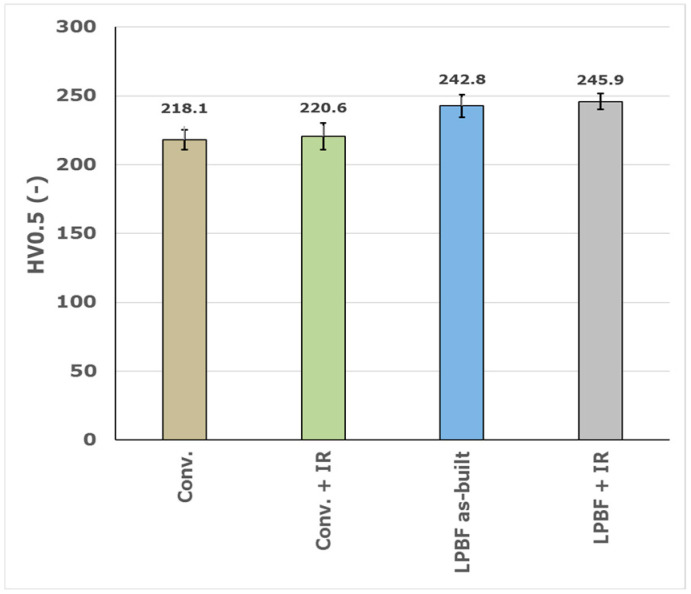
Vickers microhardness HV0.5 of conventionally manufactured (Conv.) and LPBF-produced AISI 316L samples in the uncoated and HiPIMS TiAlSiN-coated states. Error bars represent the standard deviation.

The Vickers microhardness HV0.5 (Figure 1) was evaluated on conventionally manufactured (Conv.) AISI 316L and additively manufactured samples produced by the LPBF process in the as-built and HiPIMS TiAlSiN-coated states. The conventionally manufactured material in the uncoated condition exhibited an average microhardness of 218.1 HV0.5. After coating deposition, the microhardness slightly increased to 220.6 HV0.5.

The LPBF-produced material showed higher microhardness compared to the conventionally manufactured counterpart. The LPBF as-built samples reached an average microhardness of 242.8 HV0.5, while the application of the HiPIMS TiAlSiN coating resulted in a further increase to 245.9 HV0.5. The differences in microhardness between the coated and uncoated states were relatively small for both manufacturing routes, indicating a limited influence of the coating on the measured bulk microhardness under the applied load.

Overall, the results demonstrate that the LPBF process leads to higher microhardness values compared to the conventionally manufactured material, while the presence of the coating causes only a minor increase in the microhardness measured.

### 3.2. Static Tensile Test

**Figure 2:**
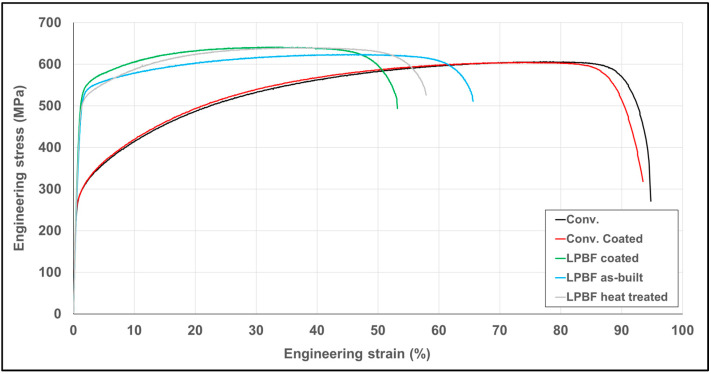
Representative engineering stress–strain curves obtained from tensile tests of conventionally manufactured and LPBF-produced samples in the as-built, heat-treated (500 °C/4.5 h), and HiPIMS TiAlSiN-coated states.

Figure 2 shows representative engineering stress–strain curves obtained from static tensile tests of conventionally manufactured and LPBF-produced samples in both uncoated and HiPIMS TiAlSiN-coated states. The conventionally manufactured material exhibited the lowest YS and the highest ductility, with TE exceeding 75%, while the application of the coating resulted in a slight increase in YS without a significant change in UTS.

The LPBF as-built material showed considerably higher yield and ultimate tensile strengths compared to the conventionally manufactured counterpart, accompanied by reduced ductility. In the coated LPBF specimens, a further increase in YS from 411 MPa to 441 MPa and in UTS from 625 MPa to 636 MPa was observed. At the same time, a pronounced decrease in TE was observed, decreasing from 62% to 48.9%. The trends observed in the representative stress–strain curves are consistent with the average mechanical properties summarized in Table 2, including yield strength, ultimate tensile strength, total elongation, and reduction in area. In order to isolate the effect of thermal exposure associated with the coating process, additional tensile tests were performed on LPBF specimens subjected to a heat treatment at 500 °C for 4.5 h, followed by slow furnace cooling (60 min) without coating deposition (LPBF heat-treated).

The heat-treated LPBF specimens exhibited a yield strength of 434 ± 3 MPa and an ultimate tensile strength of 640 ± 1 MPa, which are comparable to the values measured for the coated LPBF specimens (441 ± 5 MPa and 636 ± 3 MPa, respectively). At the same time, the total elongation reached 56 ± 2.5%, which is higher than that of the coated LPBF specimens (48.9 ± 0.9%) but lower than the as-built LPBF state (62 ± 3.3%).

### 3.3. Microstructure Analysis

**Figure 3:**
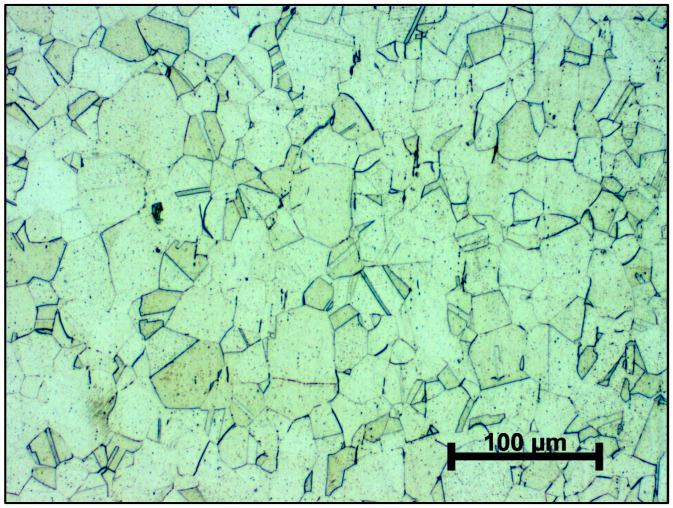
Optical micrograph of the microstructure of the conventionally manufactured AISI 316L material after heat treatment.

The optical micrograph of the conventionally manufactured AISI 316L material (Figure 3) reveals a fully recrystallized microstructure with polyhedral grains of predominantly equiaxed morphology. The grain boundaries are well defined, and the grain size appears relatively uniform across the observed area. Annealing twins, which are characteristic of austenitic stainless steels after recrystallization heat treatment, are frequently observed. No pronounced texture or preferential grain orientation is evident in the microstructure.

**Figure 4:**
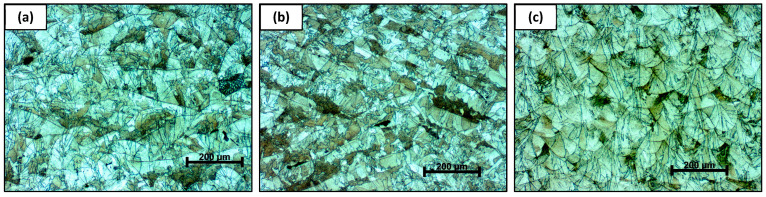
Optical micrographs of the microstructure of additively manufactured AISI 316L observed in different build orientations: (**a**) 45° orientation; (**b**) XY plane; (**c**) Z direction.

The optical micrographs of the additively manufactured AISI 316L material reveal microstructural features characteristic of the LPBF process, with a pronounced dependence on the observation plane relative to the build orientation (Figure 4). In the 45° orientation (Figure 4a), the microstructure exhibits inclined and elongated melt pool boundaries, resulting from the intersection of the observation plane with the curved melt pool geometry. This orientation leads to a mixed appearance of features typical of both vertical and horizontal sections, producing a more complex and heterogeneous microstructural pattern. The microstructure observed in the XY plane (Figure 4b) is characterized by relatively flat and elongated melt pool tracks, oriented predominantly parallel to the laser scanning direction. The melt pool boundaries appear more continuous and laterally extended, reflecting the layer-wise scanning strategy and the overlap of adjacent scan tracks within a single layer. In contrast, the microstructure observed in the Z direction (Figure 4c) shows arc-shaped and overlapping melt pool boundaries stacked along the build direction. This morphology directly reflects the layer-by-layer nature of the LPBF process, where successive melt pools solidify on top of previously deposited layers.

Across all orientations, the presence of melt pool boundaries dominates the observed microstructure, indicating that the material remained in the as-built condition without subsequent recrystallization heat treatment. The differences in melt pool morphology among the investigated orientations highlight the inherent anisotropy introduced by the LPBF process, which can influence the mechanical response of the material.

**Figure 5:**
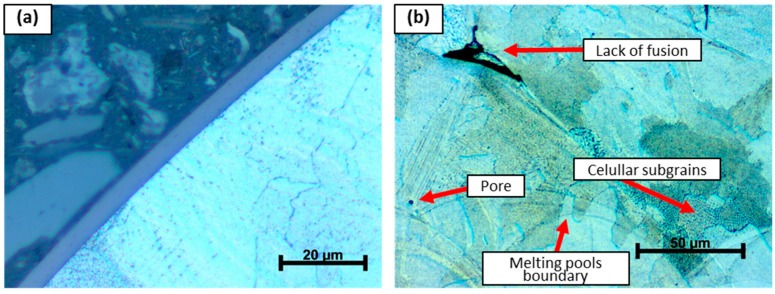
Optical micrographs of additively manufactured AISI 316L: (**a**) cross-sectional view of the sample surface in the XY direction; (**b**) representative microstructure of LPBF-manufactured material with highlighted characteristic microstructural features.

Figure 5a shows a cross-sectional view of the surface of additively manufactured AISI 316L in the XY direction, where a compact and continuous coating deposited by the HiPIMS process can be observed. The coating exhibits a uniform thickness of approximately 4.5–4.6 µm and does not show pronounced discontinuities or local delamination within the observed area, indicating a stable and well-controlled deposition process. Figure 5b presents a representative microstructure of the LPBF-produced material, in which typical microstructural features of the LPBF process are clearly visible. Distinct melt pool boundaries following the laser scanning trajectory can be identified, together with a fine cellular subgrain structure formed because of high cooling rates and steep thermal gradients during processing. Locally, isolated pores and lack-of-fusion defects are observed, originating from incomplete bonding between adjacent melt pools. These microstructural characteristics confirm that the material remained in the as-built condition, without subsequent recrystallization heat treatment.

Quantitative image analysis was performed to evaluate the defect characteristics of the LPBF-produced material. The analysis was carried out in three different section orientations (XY, Z, and 45°). The results are summarized in Table 3. The equivalent mean pore diameter ranged between 17.9 ± 7.3 µm and 22.4 ± 11.5 µm, depending on the section orientation. The largest number of detected pores, as well as the largest average pore size, were observed in the 45° section, indicating orientation-dependent defect distribution associated with the layer-by-layer nature of the LPBF process. The overall porosity of the investigated LPBF material was relatively low, reaching approximately 0.1%, which is consistent with the high-density state typically achieved in optimized LPBF processing of AISI 316L stainless steel.

### 3.4. Fractography Analysis

**Figure 6:**
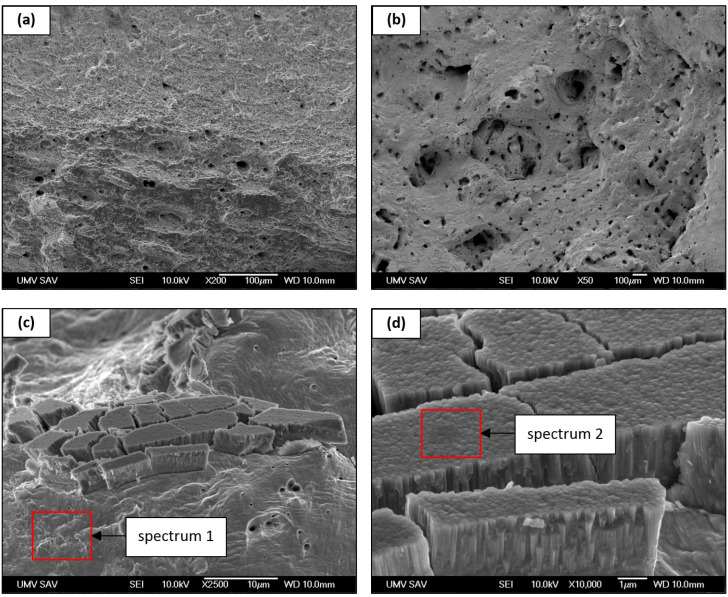
SEM fracture surface images of AISI 316L: (**a**) conventionally manufactured material; (**b**) LPBF material; (**c**) fractured HiPIMS TiAlSiN coating on the LPBF sample with EDS analysis area of the substrate (spectrum 1); (**d**) high-magnification detail of the TiAlSiN coating on the LPBF sample with EDS analysis area (spectrum 2).

Representative fractographic images of fracture surfaces of conventionally manufactured and additively manufactured AISI 316L samples, as well as details of the TiAlSiN coating deposited by the HiPIMS technique, are shown in Figure 6.

The fracture surface of the conventionally manufactured material, shown in Figure 6a, exhibits a relatively homogeneous morphology with gently undulated features and a local presence of small dimples. This appearance is characteristic of a ductile fracture mechanism in recrystallized austenitic stainless steel and corresponds well with the homogeneous microstructure observed by optical microscopy.

In contrast, the fracture surface of the additively manufactured material (Figure 6b) shows a more pronounced surface heterogeneity and an increased occurrence of cavities of various sizes. Locally, pores corresponding to internal defects formed during the LPBF process, such as lack-of-fusion voids or isolated pores, can be identified. These defects may act as local stress concentrators and influence the fracture behavior of the additively manufactured material.

A detailed fractographic image of the TiAlSiN coating deposited by the HiPIMS process (Figure 6c) documents local damage of the coating at the edges of the fracture surface. In these regions, the coating appears fragmented into segments, and the damage exhibits a predominantly brittle character, which is typical for hard PVD coatings subjected to high local mechanical loading. At the same time, it is evident that the coating remains present in the fracture region even after specimen rupture, indicating that the damage occurred preferentially within the coating itself, rather than by complete delamination from the substrate. An EDS analysis of the substrate matrix (spectrum 1) was performed directly in the region marked in Figure 6c to identify the chemical composition of the base material adjacent to the fracture. Figure 6d shows a high-magnification detail of the coating from which an EDS analysis of the TiAlSiN coating (spectrum 2) was carried out. The EDS results clearly distinguish the chemical compositions of the substrate and the coating, confirming the presence of Ti, N, Si, and Al in the coating and thus in the fracture region even after mechanical failure. These findings are consistent with fractographic observations and support the conclusion that the coating was not completely removed from the fracture area during tensile loading. The chemical compositions obtained by EDS analysis for both spectra are summarized in Table 4.

### 3.5. DSC Analysis

**Figure 7:**
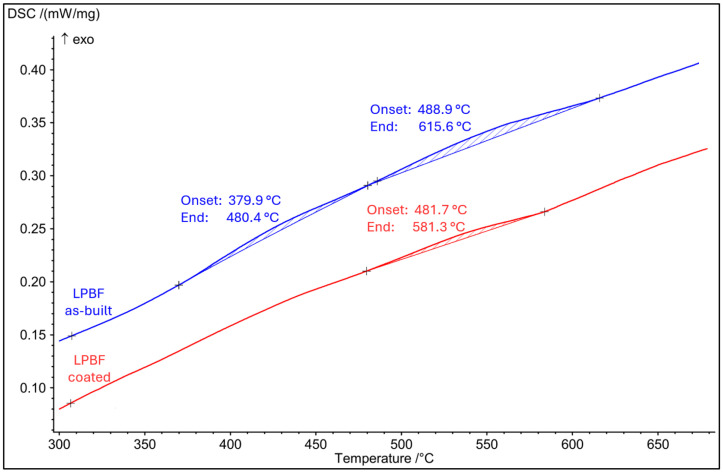
DSC curves of LPBF-produced AISI 316L stainless steel in the as-built and HiPIMS-coated conditions showing exothermic thermal responses during continuous heating.

Differential scanning calorimetry was performed to evaluate the thermal response of the LPBF-produced AISI 316L stainless steel in the temperature range relevant to the coating deposition process. The DSC curve obtained during continuous heating from room temperature to 700 °C is shown in Figure 7.

On the DSC curve of the LPBF as-built sample, two broad exothermic peaks are observed. The first peak extends over the temperature range of 379.9–480.4 °C, while the second broad exothermic event occurs between 488.9–615.6 °C. In contrast, the LPBF-coated sample exhibits only one broad exothermic peak in the interval of 481.7–581.3 °C, whereas the lower-temperature peak present in the as-built condition is no longer detected. The presence of the exothermic reaction suggests the activation of thermally driven processes in the as-built LPBF material within the temperature interval corresponding to the thermal exposure experienced during the HiPIMS coating deposition. The temperature range of the detected exothermic effect overlaps with the maximum substrate temperature reached during coating deposition (approximately 500 °C), indicating that thermally activated microstructural rearrangements may occur during this processing step.

Although DSC does not provide direct microstructural information, the observed exothermic response may be associated with relaxation and diffusion-controlled processes commonly reported for additively manufactured austenitic stainless steels. These processes are associated with the high defect density, supersaturated solid solution, and pronounced microsegregation characteristic of the as-built LPBF microstructure.

### 3.6. Crater Ball Method (Calotest)

**Figure 8:**
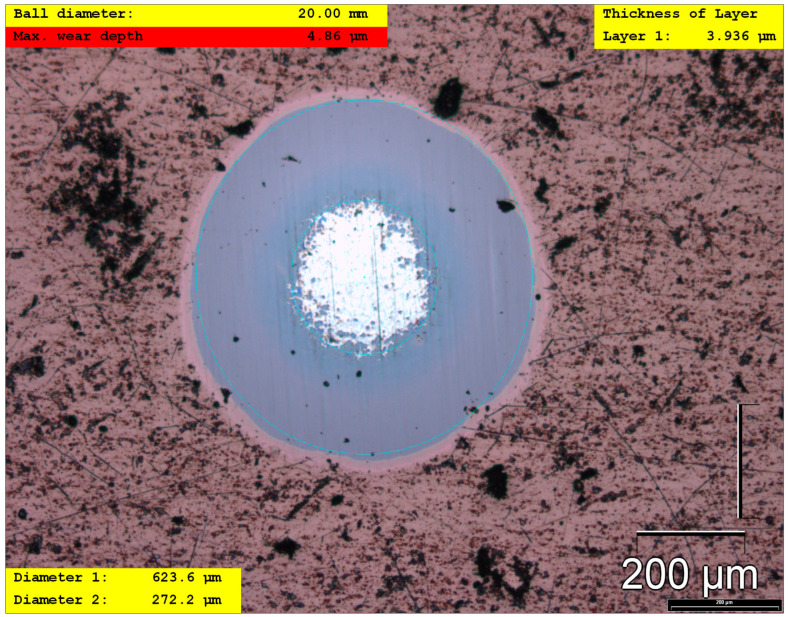
Representative wear calotte produced by the Calotest method on a HiPIMS TiAlSiN-coated sample.

The coating thickness was determined using the crater ball (Calotest) method (Figure 8). The average total coating thickness was 4.47 µm, with a maximum deviation of 0.62 µm, indicating good thickness uniformity across the coated surface. The calotte geometry exhibited a regular and symmetric wear crater, with clearly distinguishable inner and outer diameters corresponding to the coating and substrate regions, confirming reliable thickness determination and uniform layer growth. The wear calotte exhibited regular geometry with clearly defined coating–substrate boundaries, indicating uniform layer growth and stable coating cohesion. The uniform thickness distribution and controlled wear response are consistent with the high critical load for adhesive failure obtained from the scratch test, collectively confirming the good mechanical integrity and tribological stability of the HiPIMS-deposited coating.

### 3.7. Scratch Test

Scratch tests were performed to evaluate the mechanical and tribological response of the surface and, in the case of coated samples, the integrity of the coating–substrate system. The evolution of the coefficient of friction (COF) as a function of the applied normal load was continuously recorded and correlated with post-test microscopic observations of the scratch tracks.

#### 3.7.1. Uncoated States

**Figure 9:**
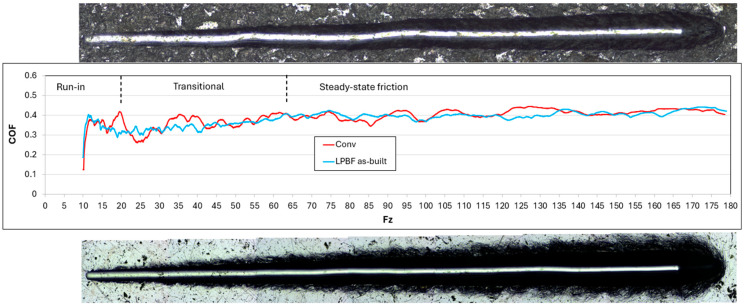
COF–F_z_ curves from progressive scratch tests of uncoated conventionally manufactured (red) and LPBF-produced (blue) AISI 316L, with indicated loading regimes (run-in, L1–L3) and corresponding scratch track morphology.

After the initial contact between the indenter and the material surface, the friction response can be divided into three characteristic regimes (Figure 9).

Region I (run-in stage, up to approximately 20 N) is characterized by a rapid increase in the coefficient of friction (COF) to approximately 0.38–0.40, followed by a short-term decrease to about 0.30–0.33 as the contact conditions stabilize. This behavior corresponds to the establishment of the real contact area and surface adaptation, including the removal or deformation of surface asperities. Optical observations within this load range did not reveal crack formation or localized surface damage for either material condition.

Region II (transitional regime, in the range of approximately 20–60 N) exhibits moderate COF fluctuations accompanied by a gradual increase toward approximately 0.38–0.40. These variations are attributed to evolving contact conditions and progressive plastic deformation rather than discrete failure events. Scratch track morphology indicates continuous groove formation with increasing material pile-up and no evidence of crack initiation. Within this regime, a slight reduction in the average COF and fluctuation amplitude was observed for the LPBF-produced AISI 316L compared to the conventionally manufactured counterpart. Although the difference is moderate, it suggests a somewhat more stable friction response of the additively manufactured material under increasing normal load.

Region III (steady-state friction, above approximately 60 N up to the maximum applied load) is characterized by relatively stable COF values in the range of approximately 0.38–0.42. No abrupt drops or sharp increases in friction were detected. Analysis of the scratch tracks confirmed stable plastic deformation behavior without lateral or radial cracking for both the conventionally manufactured and LPBF-produced AISI 316L samples.

#### 3.7.2. Coated States

**Figure 10:**
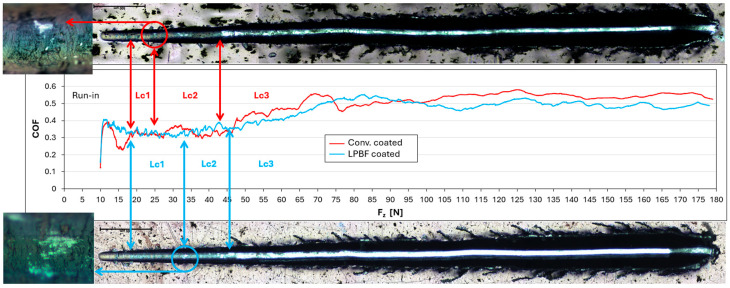
COF–F_z_ curves obtained from progressive load scratch tests of TiAlSiN-coated AISI 316L. Red curve: conventionally manufactured substrate; blue curve: LPBF substrate. Critical loads (Lc1–Lc3) are indicated.

The scratch test was performed to compare the mechanical response and damage behavior of the PVD coating deposited under identical technological conditions on conventionally manufactured and additively manufactured AISI 316L substrates. Based on the evolution of the coefficient of friction (COF) as a function of normal load (F_z_) and on the morphological analysis of the scratch track, characteristic stages of coating damage were identified (Figure 10).

At the beginning of the test, a run-in region corresponding to contact stabilization was observed up to F_z_ = 14 ± 0.5 N for the conventionally manufactured substrate and F_z_ = 16 ± 0.6 N for the additively manufactured substrate.

Three characteristic critical loads (Lc1–Lc3) were identified from the combined evaluation of COF evolution and scratch track morphology. The first critical load, Lc1, defined as the first optically observable local coating spallation, occurred at F_z_ = 18 ± 1 N for both the conventionally manufactured and additively manufactured substrates. At this stage, isolated local defects were formed without extensive coating delamination.

The second critical load, Lc2, corresponding to the onset of progressive coating delamination and repeated adhesive defects along the scratch track, was determined at F_z_ = 25 ± 0.8 N for the conventionally manufactured substrate and F_z_ = 33 ± 0.5 N for the additively manufactured substrate. Between Lc1 and Lc2, the COF remained in a quasi-steady regime with minor fluctuations, while localized cracking and isolated adhesive defects were progressively observed along the scratch path.

The third critical load, Lc3, characterized by advanced coating failure with extended delamination and frequent local substrate exposure, was identified at F_z_ = 43 ± 0.7 N for the conventionally manufactured substrate and F_z_ = 46 ± 0.8 N for the additively manufactured substrate.

Above Lc3, the COF continued to increase before transitioning into a higher quasi-stable regime at elevated loads. Overall, the additively manufactured substrate exhibited a shift in Lc2 toward higher normal loads and a more stable friction response in the post-critical regime, indicating improved mechanical support of the coating–substrate system under progressive loading.

## 4. Discussion

### 4.1. Material Characterization

The results of mechanical testing and microstructural analysis clearly demonstrate a pronounced influence of the manufacturing route on the mechanical response of AISI 316L stainless steel. This influence is quantitatively evident when comparing the conventionally manufactured material with the LPBF-produced material in the as-built condition, with differences manifested not only in strength levels but also in plastic properties.

The conventionally manufactured material reached a YS of 257 MPa and a UTS of 606 MPa, while exhibiting very high plasticity, characterized by a TE of 77.5% and a RA of 76.7%. These values are typical for solution-annealed austenitic AISI 316L and correspond well with its microstructure composed of equiaxed grains with a low dislocation density. Such a microstructural state promotes homogeneous plastic deformation and delayed strain localization, which is directly reflected in the exceptionally high ductility of the material. These values are consistent with commonly reported properties of solution-annealed wrought 316L, where YS typically lies in the range of approximately 200–300 MPa with elongation frequently above 60–70% [38,39].

In contrast, the LPBF-produced material in the as-built condition exhibited a significantly higher strength level. The YS increased to 411 MPa, representing an increase of approximately 60% compared to the conventionally manufactured material, while the UTS rose to 625 MPa. This substantial increase in strength was accompanied by a reduction in plastic properties, with TE decreasing to 62% and RA to 62.4%. This shift in the combination of mechanical properties clearly documents the typical strength–ductility trade-off characteristic of LPBF materials. A similar strength–ductility shift has been widely reported for as-built LPBF 316L, typically attributed to the fine cellular/solidification substructure and high dislocation density introduced by rapid solidification [38]. Reported as-built LPBF 316L YS in the approximately 400 MPa range, with reduced ductility compared to wrought reference states, are consistent with the trend observed in these studies [40,41,42].

The increased strength of the LPBF material is in direct agreement with its microstructural state. A fine cellular substructure with submicrometre-sized cells, together with a high dislocation density and microsegregation of alloying elements along cell boundaries, creates effective barriers to dislocation motion. This strengthening mechanism is quantitatively reflected by the increase in microhardness, which reached 242.8 HV0.5 in the LPBF as-built condition, compared to only 218.1 HV0.5 for the conventionally manufactured material. The increase in substrate microhardness, therefore, correlates well with the pronounced increase in YS and confirms the dominant role of microstructural strengthening induced by the LPBF process.

The coating process carried out at a temperature of approximately 500 °C for a total duration of 4–5 h represents a significant thermomechanical exposure for the LPBF material, which can influence both its microstructural state and mechanical properties. This temperature is sufficiently high to promote partial relaxation of macroscopic residual stresses generated during rapid solidification in the LPBF process, while remaining well below the threshold required for extensive recrystallization or the dissolution of the fine cellular substructure characteristic of the as-built condition. Stress-relief treatments and subcritical thermal exposures of LPBF 316L in the range of approximately 450–600 °C have been shown to reduce residual stresses while retaining much of the as-built microstructural character when recrystallization temperatures are not reached [43]. The retention of the main strengthening features of this substructure is further supported by the absence of any decrease in microhardness or strength after coating deposition; instead, a slight increase in these properties was observed. Such behavior is consistent with reports that subcritical heat exposure may activate recovery/relaxation phenomena in LPBF metals without necessarily causing bulk softening when the strengthening substructure is preserved [43,44].

From a mechanical perspective, thermal exposure during coating deposition was associated with a slight increase in YS from 411 MPa to 441 MPa, accompanied by a slight increase in UTS, while the microhardness increased only to a limited extent. This behaviour suggests that thermal exposure during coating did not result in material softening and may be associated with recovery-type processes, defect rearrangement or stress relaxation. In addition, thermally activated phenomena such as dynamic strain aging or the formation of nanoscale precipitates may occur in austenitic stainless steels exposed to temperatures around 500 °C and could contribute to the observed increase in strength accompanied by a reduction in ductility [45]. Selective relaxation of internal residual stresses may have enabled a more uniform distribution of plastic deformation during the early stages of loading, thereby increasing the effective YS. The absence of microhardness reduction after coating deposition suggests that the main strengthening contribution of the cellular substructure was retained despite thermal exposure. Additional tensile tests performed on LPBF specimens subjected to the same thermal cycle as the coating process (500 °C for 4.5 h) without coating deposition provide further insight into the role of thermal exposure. The mechanical response of the heat-treated LPBF specimens was similar to that of the coated LPBF condition in terms of strength, while the ductility remained higher than in the coated state. This comparison indicates that thermal exposure contributes to the observed increase in strength, whereas the additional reduction in ductility in the coated specimens may also be associated with surface-related effects such as strain localization or crack initiation at the coating–substrate interface [46]. The reduction in plastic properties after coating deposition, manifested by a decrease in TE from 62% to 48.9%, cannot be attributed solely to the thermal effect of the coating process. The comparison with LPBF specimens subjected to the same thermal cycle without coating deposition indicates that thermal exposure contributes to the observed change in tensile response; however, the lower ductility of the coated specimens suggests that additional surface-related effects may also be involved. Given the relatively small thickness of the TiAlSiN coating (approximately 4.5 µm) compared to the cross-sectional dimensions of the tensile specimens, the observed changes in mechanical properties cannot be associated with a direct load-bearing contribution of the coating. Instead, they may reflect the combined influence of deposition-related thermal exposure and local surface effects, such as strain localization or crack initiation at the coating–substrate interface. Since no direct residual stress or in situ crack-initiation measurements were performed, these mechanisms should be considered as possible contributing factors rather than directly proven causes.

Fractographic analysis supports these observations. The conventionally manufactured material exhibited a predominantly ductile fracture with uniformly distributed dimples, whereas the LPBF material showed a more heterogeneous fracture surface. Locally enlarged dimples and fracture initiation sites were associated with microstructural heterogeneities and process-induced defects, which is consistent with the observed reduction in plastic properties. Given the low measured pore area fraction (~0.1%), porosity was not considered a dominant factor controlling the overall mechanical behaviour. However, isolated process-related defects may still have acted locally as stress concentrators and contributed to crack or fracture initiation in LPBF specimens. Similar fracture behaviour of LPBF-produced AISI 316L has been repeatedly reported in the literature, where the as-built condition is characterized by high strength combined with a limited ductility reserve compared to conventionally processed material.

### 4.2. Suitability of LPBF AISI 316L for HiPIMS TiAlSiN Coating

From the perspective of coated systems, the substrate plays a critical role in determining the mechanical integrity and damage response of the coating–substrate assembly. In coated systems subjected to progressive scratch loading, the mechanical response of the substrate under contact stress governs stress redistribution within the coating–substrate assembly and influences the onset of interfacial shear-driven failure. As demonstrated in Section 4.1, AISI 316L produced by the LPBF process exhibits a higher load-bearing capacity compared to its conventionally manufactured counterpart, resulting from its refined microstructure and elevated strength. These characteristics are particularly relevant for hard PVD coatings, where the ability of the substrate to support the coating under contact loading governs the onset and progression of coating damage. It should be noted that the mechanical response of the coating–substrate system during scratch testing is influenced not only by the intrinsic properties of the coating but also by the load-bearing capacity of the substrate. Since the TiAlSiN coating in this study was deposited under identical HiPIMS conditions for both substrate types, the intrinsic coating properties are expected to be comparable. Therefore, the differences observed in the critical loads are primarily attributed to the different mechanical responses of the substrates.

The scratch test results indicate that the LPBF-produced substrate provides more effective mechanical support for the HiPIMS TiAlSiN coating. In the coated state, the LPBF substrate exhibits a more stable evolution of the coefficient of friction during the initial and intermediate loading regimes (L1 and L2), accompanied by reduced oscillations in the COF signal. Although the first critical load (Lc1) was comparable for both substrates, the second critical load (Lc2) shifted from approximately 25 N for the conventional substrate to approximately 33 N for the LPBF substrate. Since Lc2 corresponds to the onset of progressive coating delamination, this shift suggests delayed development of adhesive damage under increasing normal load. This behavior may indicate a more uniform stress transfer from the coating to the substrate and a delayed transition from elastic–plastic deformation of the coating to adhesive damage. In contrast, coatings deposited on the conventionally manufactured substrate show an earlier onset of coating degradation and a more pronounced increase in COF at comparable normal loads.

The enhanced load-bearing capacity of the LPBF substrate is primarily reflected in the extension of the L2 regime toward higher normal forces before the onset of progressive adhesive damage. While Lc1 and Lc3 were comparable for both substrates, the most pronounced difference was observed in Lc2, corresponding to the onset of progressive delamination. This indicates improved intermediate load-bearing support rather than a substantial difference in ultimate adhesion strength. Mechanistically, this response can be associated with reduced plastic deformation beneath the indenter, which limits the formation of stress concentrations at the coating–substrate interface and decreases the driving force for crack propagation. This trend agrees with classical scratch-adhesion observations showing that the measured critical load often increases with increasing substrate hardness due to improved load-bearing capacity [47].

The results obtained from the Calotest further support these findings. The HiPIMS TiAlSiN coating exhibited a uniform thickness distribution and a regular, symmetric wear calotte without signs of premature delamination or irregular spallation. The measured average coating thickness of 4.47 μm with a maximum deviation of 0.62 μm confirms homogeneous coating growth and consistent deposition conditions across the tested substrates. The well-defined coating–substrate interface and the absence of coating failure are consistent with the high critical loads observed in the scratch tests, confirming the good adhesion and mechanical stability of the coating deposited on the LPBF substrate.

It is also important to consider the thermal exposure associated with the HiPIMS deposition process, which was carried out at temperatures up to approximately 500 °C. This temperature is sufficient to promote partial relaxation of residual stresses inherent to LPBF-produced materials, while remaining below the threshold required for significant recrystallization or degradation of the fine cellular substructure. Consequently, the LPBF substrate is expected to retain its load-bearing capacity after coating deposition, which is consistent with the absence of any reduction in critical loads observed during scratch testing. Recent reviews of PVD adhesion mechanisms emphasize that scratch-test critical loads are strongly influenced by the substrate’s mechanical support, residual stresses, and the coating–substrate interfacial integrity, which aligns with the present interpretation of the LPBF substrate effect [48,49,50].

Overall, the combined results indicate that LPBF-produced AISI 316L represents a promising substrate for HiPIMS-deposited TiAlSiN coatings under progressive contact loading.

## 5. Conclusions

This study demonstrates that the manufacturing route of AISI 316L significantly influences the mechanical support and scratch-damage response of HiPIMS-deposited TiAlSiN coatings. The refined cellular microstructure of LPBF-produced 316L enhances the load-bearing capacity of the substrate and improves the mechanical response of the coating–substrate system under progressive contact loading.

The main conclusions are:

1. LPBF-produced AISI 316L exhibited significantly higher yield strength (411 MPa vs. 257 MPa) and microhardness (242.8 HV0.5 vs. 218.1 HV0.5) compared to conventionally manufactured material, which can be attributed to the refined cellular microstructure formed during rapid solidification.
2. Thermal exposure during the HiPIMS deposition process (500 ± 10 °C, 4–5 h) did not indicate substrate softening under the applied test conditions. Instead, a slight increase in yield strength to 441 MPa was observed, while microhardness remained essentially unchanged.
3. The TiAlSiN coating exhibited a uniform thickness of 4.47 ± 0.62 µm and a well-defined coating–substrate interface, with no visible signs of premature delamination in the performed observations.
4. Scratch testing revealed that the LPBF-produced substrate provides improved mechanical support for the coating. In particular, the onset of progressive coating delamination (Lc2) increased from approximately 25 N for the conventionally manufactured substrate to approximately 33 N for the LPBF substrate, corresponding to an increase of about 32%.
5. Although ductility decreased after coating deposition (62% → 48.9%), the coated LPBF system demonstrated improved resistance to progressive adhesive damage under increasing contact load.

## Figures and Tables

**Table 1:** Process parameters used for LPBF fabrication of AISI 316L samples.

Parameter	Value	Unit
Laser power	200	W
Scan speed	650	mm·s^−1^
Layer thickness	0.05	mm
Hatching distance	0.11	mm
Laser spot size	0.075	mm
Volumetric energy density	56	J·mm^−3^
Inert gas	Argon	

**Table 2:** Average tensile properties obtained from static tensile tests of conventionally manufactured and LPBF-produced samples in the uncoated and HiPIMS TiAlSiN-coated states.

State	YS (MPa) ± Dev	UTS (MPa) ± Dev	TE (%) ± Dev	RA (%) ± Dev
Conv.	257 ± 3	606 ± 2	77.5 ± 1.8	76.7 ± 0.8
Conv. coated	270 ± 5	605 ± 1	76.4 ± 1.1	75.1 ± 1.4
LPBF as-built	411 ± 4	625 ± 1	62 ± 3.3	62.4 ± 2.4
LPBF-coated	441 ± 5	636 ± 3	48.9 ± 0.9	59.9 ± 2.1
LPBF heat-treated	434 ± 3	640 ± 1	56 ± 2.5	61.3 ± 3.1

**Table 3:** Quantitative evaluation of pore characteristics in LPBF-produced AISI 316L based on optical micrograph image analysis performed in three section orientations (XY, Z and 45°).

Section Orientation	Number of Analyzed Pores	Mean Pore Area (µm^2^)	Eqv. Mean Pore Diameter (µm)
XY	57	251.8	17.9 ± 7.3
Z	62	271.8	18.6 ± 10.1
45°	112	393.4	22.4 ± 11.5

**Table 4:** Chemical composition determined by EDS analysis for the substrate matrix (spectrum 1) and the TiAlSiN coating (spectrum 2), expressed in weight percentages.

Spectrum	Element	Weight (%)
1—Substrate (matrix)	Fe	68.14
	Cr	18.07
	Ni	10.55
	Mo	2.58
	Si	0.67
2—TiAlSiN coating	Ti	58.58
	N	31.21
	Si	8.35
	Al	1.86

## Data Availability

The original contributions presented in this study are included in the article. Further inquiries can be directed to the corresponding authors.
